# Tracheal Intubation during Advanced Life Support Using Direct Laryngoscopy versus Glidescope^®^ Videolaryngoscopy by Clinicians with Limited Intubation Experience: A Systematic Review and Meta-Analysis

**DOI:** 10.3390/jcm11216291

**Published:** 2022-10-26

**Authors:** Hans van Schuppen, Kamil Wojciechowicz, Markus W. Hollmann, Benedikt Preckel

**Affiliations:** Department of Anesthesiology, Amsterdam UMC location University of Amsterdam, Meibergdreef 9, 1105 AZ Amsterdam, The Netherlands

**Keywords:** airway management, cardiopulmonary resuscitation, advanced life support, emergency medical service, tracheal intubation, videolaryngoscopy

## Abstract

The use of the Glidescope^®^ videolaryngoscope might improve tracheal intubation performance in clinicians with limited intubation experience, especially during cardiopulmonary resuscitation (CPR). The objective of this systematic review and meta-analysis is to compare direct laryngoscopy to Glidescope^®^ videolaryngoscopy by these clinicians. PubMed/Medline and Embase were searched from their inception to 7 July 2020 for randomized controlled trials, including simulation studies. Studies on adult patients or adult-sized manikins were included when direct laryngoscopy was compared to Glidescope^®^ videolaryngoscopy by clinicians with limited experience in tracheal intubation (<10 intubations per year). The primary outcome was the intubation first-pass success rate. Secondary outcomes were time to successful intubation and chest compression interruption duration during intubation. The risk of bias was assessed with the Cochrane risk of bias tool. Certainty of evidence was assessed using the Grading of Recommendations Assessment, Development and Evaluation (GRADE). We included 4 clinical trials with 525 patients and 20 manikin trials with 2547 intubations. Meta-analyses favored Glidescope^®^ videolaryngoscopy over direct laryngoscopy regarding first-pass success (clinical trials: risk ratio [RR] = 1.61; 95% confidence interval [CI]: 1.16–2.23; manikin trials: RR = 1.17; 95% CI: 1.09–1.25). Clinical trials showed a shorter time to achieve successful intubation when using the Glidescope^®^ (mean difference = 17.04 s; 95% CI: 8.51–25.57 s). Chest compression interruption duration was decreased when using the Glidescope^®^ videolaryngoscope. The certainty of evidence ranged from very low to moderate. When clinicians with limited intubation experience have to perform tracheal intubation during advanced life support, the use of the Glidescope^®^ videolaryngoscope improves intubation and CPR performance compared to direct laryngoscopy.

## 1. Introduction

Airway management is an essential part of advanced life support to facilitate ventilation of the lungs. During cardiopulmonary resuscitation (CPR), many professionals still favor tracheal intubation, although supraglottic airway devices (SAD) are increasingly used as the primary advanced airway technique. However, when SADs fail to facilitate oxygenation in situations such as aspiration, drowning, or trauma, tracheal intubation is still indicated. In addition, in the current COVID-19 pandemic tracheal intubation is regarded as the best airway technique during CPR to minimize aerosol generation by chest compressions [1,2].

The challenge with tracheal intubation during CPR is to achieve first-pass success, while fast, safe, and without interruption of chest compressions. More experienced clinicians have a higher intubation success rate but gaining and maintaining sufficient experience in tracheal intubation is challenging, especially for EMS organizations [3,4,5]. It takes a minimum of 50 tracheal intubations to achieve an intubation success rate of 90% within two attempts, under optimal (non-emergency) conditions [6]. More than 240 tracheal intubations are needed to perform tracheal intubation during CPR with a 90% success rate and high-quality standards [7]. EMS clinicians often perform less than 10 tracheal intubations per year [8,9]. Getting a clinician with sufficient intubation experience on the scene within an acceptable time is often a challenge and sometimes not possible, such as in remote and military settings. There are several risks when tracheal intubation is performed by personnel with limited experience, including oropharyngeal injury, significant interruption of chest compressions, and incorrect tube placement with consecutive hypoxia [10,11,12].

**Figure 1:**
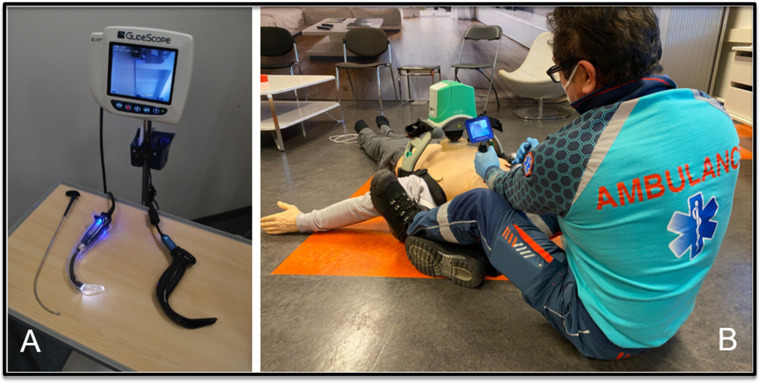
(**A**) The Glidescope^®^ videolaryngoscope with the former GVL blade (**left**) and recent LoPro blade (**right**), and the Gliderite^®^ rigid stylet. (**B**) The portable GlidescopeGo^®^, used by an EMS clinician in a simulated prehospital advanced life support setting.

Videolaryngoscopy has the potential to increase the tracheal intubation success rate when clinicians with limited experience are confronted with a patient with an indication for tracheal intubation. Furthermore, videolaryngoscopy may decrease interruptions in chest compressions during CPR. The Glidescope^®^ was the first commercially available videolaryngoscope. The hyperangulated blade includes a camera, connected to a video screen, which improves visualization of the larynx (Figure 1). The tracheal tube can then be inserted into the airway by using a rigid stylet (Gliderite^®^ Stylet).

The objective of this study was to perform a systematic review and meta-analysis on the use of the hyperangulated Glidescope^®^ videolaryngoscope for tracheal intubation by clinicians with limited intubation experience regarding first-pass success rate and time to intubation when compared to direct laryngoscopy. The secondary aim was to determine differences in chest compression interruptions during CPR. As the Glidescope^®^ videolaryngoscope is one of the most widely used videolaryngoscopes and prehospital care clinicians often have an annual tracheal intubation exposure of <10 tracheal intubations we searched for studies comparing direct laryngoscopy to Glidescope^®^ videolaryngoscopy in oral tracheal intubation by clinicians with limited experience in tracheal intubation. With this study we aimed to provide an answer to the question; should clinicians with limited intubation experience use the Glidescope^®^ for tracheal intubation?

## 2. Materials and Methods

The protocol of this systematic review and meta-analysis has been registered in the international prospective register of systematic reviews PROSPERO (review record CRD42018096251) and is included in Supplementary Materials 1. This review followed the Preferred Reporting Items for Systematic Reviews and Meta-Analyses (PRISMA) guidelines, see Supplementary Materials 2 for the PRISMA checklist [13]. Primary outcome is first-pass success rate of tracheal intubation, secondary outcomes are time needed for successful intubation and duration of chest compression interruption for CPR.

### 2.1. Eligibility Criteria

Studies were included if they met all of the following criteria:

1. Comparison of direct laryngoscopy to Glidescope^®^ videolaryngoscopy (either conventional Glidescope^®^ or Glidescope^®^ Ranger) for tracheal intubation
2. Randomized and quasi-randomized controlled trials
3. Clinicians had limited experience in tracheal intubation, defined as less than 10 intubations per year
4. Adult patients or adult-sized manikins
5. Contained any outcome of interest (first-pass success rate, and/or time to intubation, and/or hands-off time during CPR)

Studies on nasotracheal intubation were excluded.

### 2.2. Information Sources and Search Strategy

MEDLINE/PubMed and Embase were systematically searched (1966 to 7 July 2020) for randomized trials comparing tracheal intubation using direct laryngoscopy versus Glidescope^®^ videolaryngoscopy. The following search terms were used in MEDLINE/PubMed: (Glidescope^®^) OR (video laryngoscop* [tiab]). In Embase the search term (Glidescope^®^ or video laryngoscop*).ti,ab,kw was used. Bibliographies of selected manuscripts were hand-searched for additional relevant studies.

### 2.3. Study Selection

The first author (KW) performed the search. In duplicate and independently, the first two authors (K.W., H.S.) performed the bibliographic review of the search results. Disagreement was resolved by discussion and arbitrated if necessary, by a third independent researcher (BP).

### 2.4. Data Collection and Data Items

Two reviewers extracted data including the year of publication, country of origin, sample size, operator background, operator training, whether intubation was performed on a real patient or manikin, in which setting the intubation was performed, rate of successful intubation at first attempt, time required to intubate, and hands-off time during CPR. We contacted investigators for missing data if necessary.

### 2.5. Risk of Bias in Individual Studies

Risk of bias in the individual studies was independently reviewed by two investigators. The Cochrane risk of bias tool was used to determine selection bias, performance bias, detection bias, attrition bias, reporting bias, and other biases in the included studies [14].

### 2.6. Data Synthesis and Analysis

Because the setting of actual patient care and differences in design of simulation studies can influence the results, the analysis was divided into three subgroups. The three main groups are:

1. Intubations performed in the clinical setting
2. Intubations performed in a simulation setting, using manikins
3. Intubations performed in a simulation setting, using manikins with a difficult airway scenario

Differences in first-pass intubation success rate are expressed in risk ratio (RR) and differences in time to intubate and hands-off time during CPR are expressed in mean difference (MD) in seconds. Interquartile ranges were converted into standard deviations. The random effects method of Mantel–Haenszel was used to generate a pooled RR or MD across studies. We assessed statistical heterogeneity using Cochrane’s Q statistic (with *p* < 0.05 considered significant) and expressed the quantity using the I2 statistic and 95% confidence interval (CI). We followed the Cochrane handbook classification for importance of I2. To explore heterogeneity, subgroup analyses were performed for specific clinical scenarios (normal airway, difficult airway, etc.). Statistical analyses as well as forest plots were made using Review Manager (RevMan) [Computer program], Version 5.2. Copenhagen: The Nordic Cochrane Centre, The Cochrane Collaboration, 2012. The overall certainty of evidence was assessed using the Grading of Recommendations Assessment, Development and Evaluation (GRADE) method [15]. The GRADE tables were made with the GRADEpro GDT online software [GRADEpro GDT: GRADEpro Guideline Development Tool [Software]. McMaster University and Evidence Prime, 2021. Available from gradepro.org, accessed on 31 January 2022].

## 3. Results

### 3.1. Study Selection

**Figure 2:**
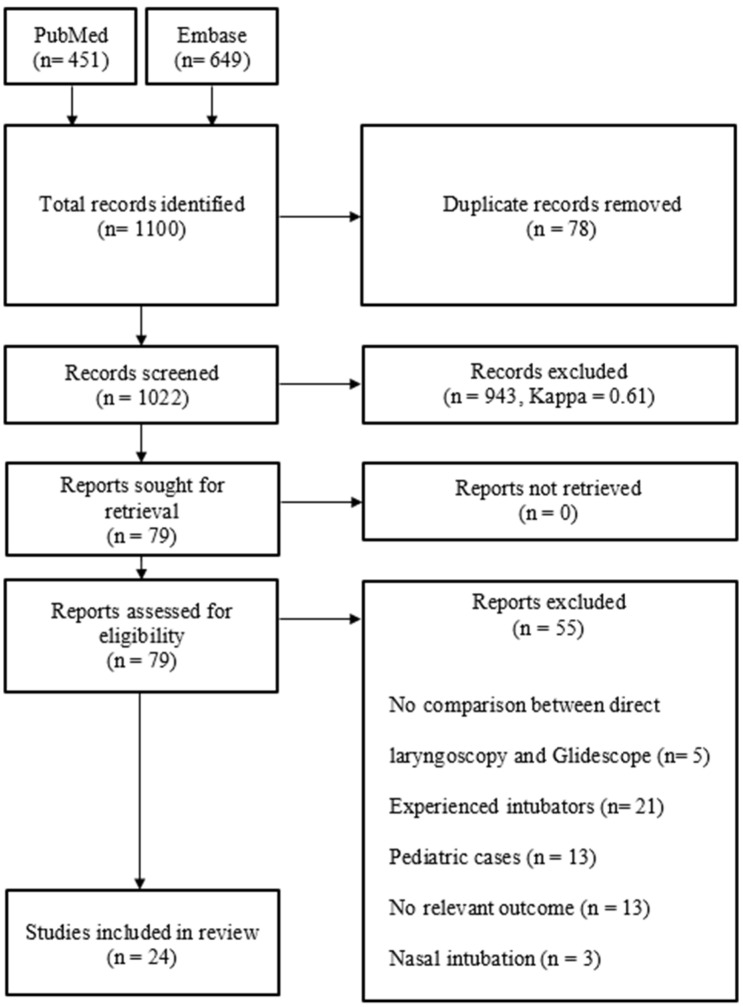
PRISMA diagram.

The literature search was performed on 7 July 2020. A total of 1022 citations were identified from Medline (PubMed) and Embase (Ovid). We excluded 943 citations on the initial screening of the title and abstract of the article and 13 on the screening of the full article. Of the remaining articles, 42 articles were excluded because there was no data on the primary or secondary endpoint, it regarded only pediatric patients, it regarded nasal intubation, there was no comparison between direct laryngoscopy and Glidescope^®^, or intubation was performed by experienced clinicians. The references of the 55 excluded articles are included in Supplementary Materials 3. Three articles were included after discussion by the first two authors. This resulted in 24 inclusions and 997 exclusions (Figure 2).

### 3.2. Study Characteristics

We identified four randomized trials in which patients were intubated in the clinical setting and 20 randomized trials in which the intubation was performed on a manikin [16,17,18,19,20,21,22,23,24,25,26,27,28,29,30,31,32,33,34,35,36,37,38,39]. See Table 1 for the study characteristics. A total of 525 clinical intubations were included as well as 2547 manikin intubations. Of the clinical studies, three were performed on patients in the operating room (OR) and one was performed during CPR. The patients included in the OR setting were ASA 1 and 2 patients in elective situations, without a known or anticipated difficult airway.

Of the manikin studies, nine studies had a protocol with only a normal airway, eleven studies included a protocol with a manikin with a normal airway as well as a difficult airway, and one study solely included a protocol with a manikin with a difficult airway. Difficult airway protocols included cervical spine immobilization, intubation during chest compressions, tongue edema or pharyngeal obstruction, a Cormack–Lehane grade 3 view, intubation on the floor, or a combination of two or more of these circumstances. When a study allowed participants to run the same scenario more than once, the data of the first scenario was included for meta-analysis.

In the trials using a manikin, all participants used both intubation techniques. In all trials except one, the operators had no prior intubation experience. In the trial in which the operators had prior intubation experience, all operators had performed less than 10 intubations in their careers [25].

### 3.3. Certainty of Evidence across Studies

The evidence was rated as low to moderate certainty regarding first-pass success rate and as very low to moderate certainty regarding time to intubation when using Glidescope^®^ videolaryngoscopy versus direct laryngoscopy. See Supplementary Materials 4 for the GRADE table. Only one study reported chest compression interruptions [39].

### 3.4. Risk of Bias within Studies

**Figure 3:**
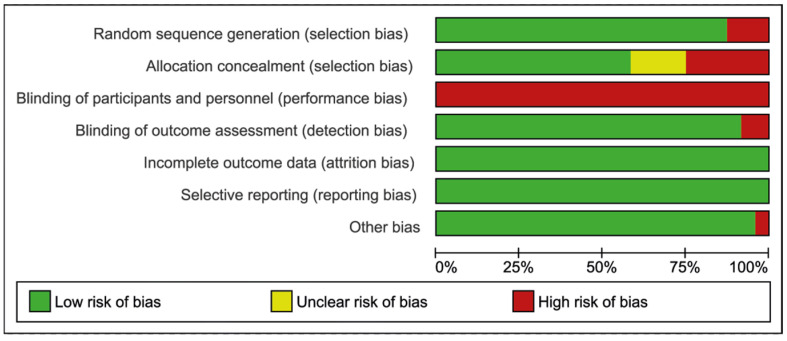
Risk of bias graph.

**Figure 4:**
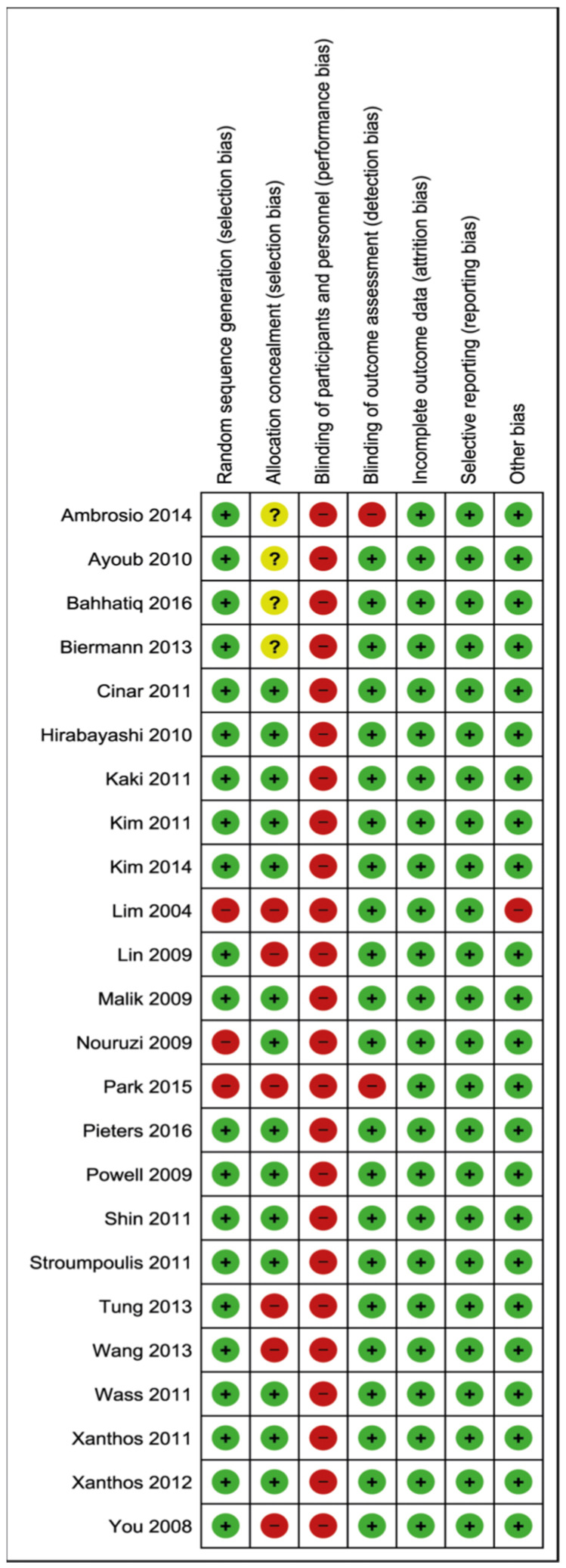
Risk of bias summary. Ambrosio, 2014 [32]; Ayoub, 2010 [36]; Bahhatiq, 2016 [34]; Biermann, 2013 [29]; Cinar, 2011 [21]; Hirabayashi, 2010 [37]; Kaki, 2011 [22]; Kim, 2011 [23]; Kim, 2014 [33]; Lim, 2004 [16]; Lin, 2009 [17]; Malik, 2009 [18]; Nouruzi, 2009 [38]; Park, 2015 [39]; Pieters, 2016 [35]; Powell, 2009 [19]; Shin, 2011 [25]; Stroumpoulis, 2011 [26]; Tung, 2013 [30]; Wang, 2013 [31]; Wass, 2011 [24]; Xanthos, 2011 [27]; Xanthos, 2012 [28]; You, 2009 [20]. Green +: low risk of bias, Red -: high risk of bias, Yellow ?: unclear risk of bias.

The risk of bias within all included studies (both clinical and manikin studies) is illustrated in Figure 3 and Figure 4 [14]. The performance bias attributes to the blinding of participants and personnel. As it is impossible to blind participants and observers for the device used, all included studies score a high risk of bias in this domain.

### 3.5. Outcomes

#### 3.5.1. Intubation First-Pass Success Rate

**Figure 5:**
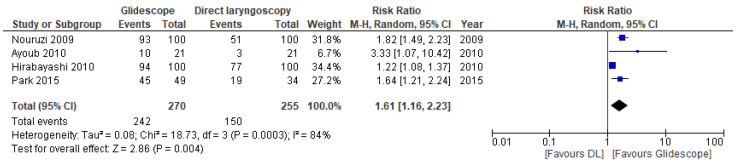
Risk ratios (RR) of successful first-attempt intubation in clinical trials comparing Glidescope^®^ videolaryngoscopy to direct laryngoscopy. The pooled estimate (rhombus) was derived using the DerSimonian and Laird random effects method. The squares depict individual study point estimates of the RR. Horizontal lines display the 95% CI of the point estimate. The vertical line represents an RR of 1.00 indicating no difference between Glidescope^®^ videolaryngoscopy and direct laryngoscopy; (DL = direct laryngoscopy). Nouruzi, 2009 [38]; Ayoub, 2010 [36]; Hirabayashi, 2010 [37]; Park, 2015 [39].

All included trials presented data on intubation success and time needed for intubation (Table 2). The pooled RR for first-pass success across the clinical studies was 1.61 (95% CI 1.16, 2.23; *p* = 0.004) (Figure 5). The level of certainty of the evidence was moderate.

**Figure 6:**
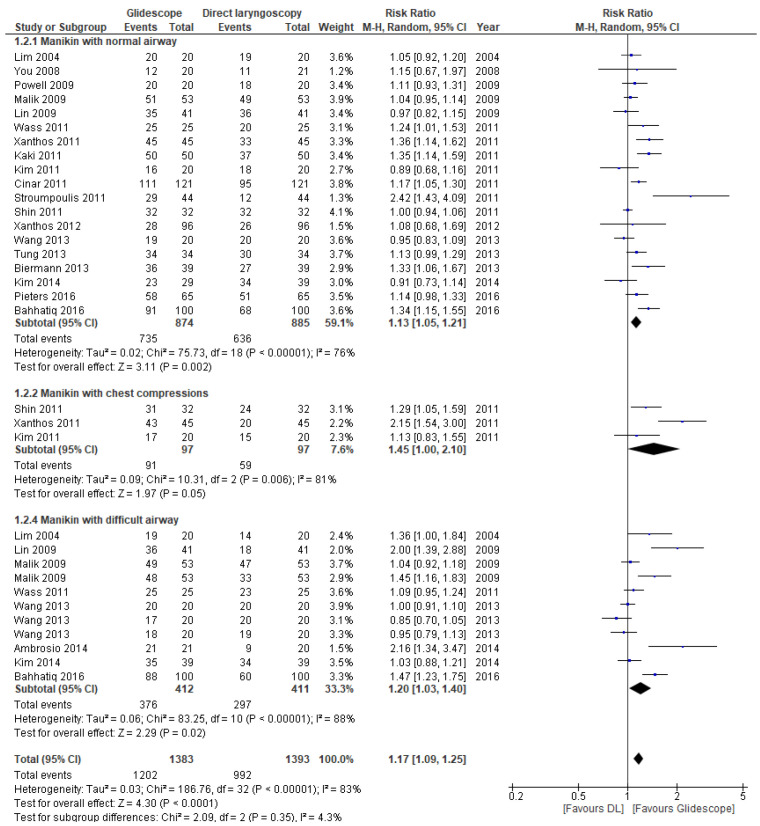
Risk ratios (RR) of successful first-attempt intubation in manikin trials comparing Glidescope^®^ videolaryngoscopy to direct laryngoscopy. The pooled estimate (rhombus) was derived using the DerSimonian and Laird random effects method. The squares depict individual study point estimates of the RR. Horizontal lines display the 95% CI of the point estimate. The vertical line represents an RR of 1.00 indicating no difference between Glidescope^®^ videolaryngoscopy and direct laryngoscopy; DL = direct laryngoscopy, GS = Glidescope^®^ videolaryngoscopy). Ambrosio, 2014 [32]; Bahhatiq, 2016 [34]; Biermann, 2013 [29]; Cinar, 2011 [21]; Kaki, 2011 [22]; Kim, 2011 [23]; Kim, 2014 [33]; Lim, 2004 [16]; Lin, 2009 [17]; Malik, 2009 [18]; Pieters, 2016 [35]; Powell, 2009 [19]; Shin, 2011 [25]; Stroumpoulis, 2011 [26]; Tung, 2013 [30]; Wang, 2013 [31]; Wass, 2011 [24]; Xanthos, 2011 [27]; Xanthos, 2012 [28]; You, 2009 [20].

The pooled RR for first-pass success in the manikin studies was 1.17 (95% CI 1.09, 1.25; *p* < 0.0001) (Figure 6). There was substantial between-study heterogeneity. All three sub-group analyses revealed a significant effect in favor of the Glidescope^®^ videolaryngoscope. The overall certainty of the evidence was rated as low for these trials, see Supplementary Materials 4 for the GRADE table.

#### 3.5.2. Time to Intubation

**Figure 7:**
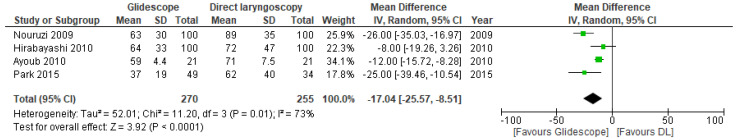
Mean difference (MD) in time to intubation (in seconds) in clinical trials comparing Glidescope^®^ videolaryngoscope to direct laryngoscopy. The pooled estimate (rhombus) was derived using the DerSimonian and Laird random effects method. The squares depict an individual study point estimate of the mean difference. Solid horizontal lines display the 95% CI of the point estimate. The vertical line represents an MD of o, indicating no difference between Glidescope^®^ videolaryngoscopy and direct laryngoscopy. (DL = direct laryngoscopy). Nouruzi, 2009 [38]; Ayoub, 2010 [36]; Hirabayashi, 2010 [37]; Park, 2015 [39].

**Figure 8:**
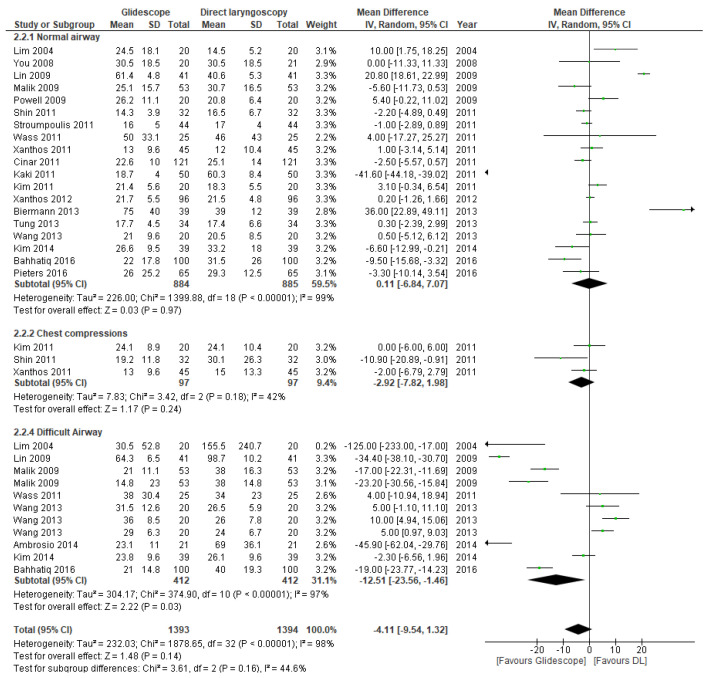
Mean difference (MD) in time to intubation (in seconds) in manikin trials comparing Glidescope^®^ videolaryngoscope to direct laryngoscopy. The pooled estimate (rhombus) was derived using the DerSimonian and Laird random effects method. The squares depict an individual study point estimate of the mean difference. Solid horizontal lines display the 95% CI of the point estimate. The vertical line represents an MD of o, indicating no difference between Glidescope^®^ videolaryngoscopy and direct laryngoscopy. (DL = direct laryngoscopy). Ambrosio, 2014 [32]; Bahhatiq, 2016 [34]; Biermann, 2013 [29]; Cinar, 2011 [21]; Kaki, 2011 [22]; Kim, 2011 [23]; Kim, 2014 [33]; Lim, 2004 [16]; Lin, 2009 [17]; Malik, 2009 [18]; Pieters, 2016 [35]; Powell, 2009 [19]; Shin, 2011 [25]; Stroumpoulis, 2011 [26]; Tung, 2013 [30]; Wang, 2013 [31]; Wass, 2011 [24]; Xanthos, 2011 [27]; Xanthos, 2012 [28]; You, 2009 [20].

The time required to intubate was available in all included studies (Table 2). The forest plots in Figure 7 and Figure 8 represent the clinical and manikin trials, respectively. The pooled MD across the clinical trials favors the Glidescope^®^ videolaryngoscope (MD 17.04 s, 95% CI 8.51, 25.57 s; *p* < 0.0001). There was substantial between-study heterogeneity, and the overall certainty of the evidence was graded as moderate. Of the manikin trials, only the subgroup analyses with the difficult airway scenarios showed a significant difference in mean intubation time in favor of the Glidescope^®^ videolaryngoscope (MD 12.51 s, 95% CI 1.46, 23.56 s; *p* = 0.03). There was considerable between-study heterogeneity. The other subgroups showed no significant difference in intubation times. The overall certainty of the evidence was rated as very low for the manikin trials, because of inconsistency and indirectness (see Supplementary Materials 4 for GRADE table).

#### 3.5.3. Intubation during Cardiopulmonary Resuscitation

We included one clinical study performed on patients during cardiac arrest [39]. The first-pass success rate of the Glidescope^®^ group was higher than that of the DL group (91.8% versus 55.9%, *p* < 0.001). It took less time to complete tracheal intubation with Glidescope^®^ than with DL (median time 37 vs. 62 s; *p* < 0.001). The median duration of chest compression interruptions during CPR was reduced from seven seconds (IQR 3–16 s) with direct laryngoscopy to zero seconds (IQR 0–0 s) with Glidescope^®^ videolaryngoscopy.

Three manikin studies reported on tracheal intubation during chest compressions [23,25,27]. The RR of successful intubation with the Glidescope^®^ was 1.45 (95% CI 1.00, 2.10; *p* = 0.05). There was substantial between-study heterogeneity. The time required for intubation was not significantly different. All three manikin studies used scenarios with uninterrupted chest compressions, so the difference in duration of chest compression interruptions was not an applicable outcome measure.

## 4. Discussion

This systematic review and meta-analysis of the literature on tracheal intubation using direct laryngoscopy versus videolaryngoscopy with the Glidescope^®^ videolaryngoscope by clinicians with limited intubation experience showed a significant improvement in first-pass success rate in both clinical and manikin randomized trials, a shorter time needed for intubation in clinical trials as well as in manikin trials with difficult airway scenarios. The only clinical trial in the CPR setting showed a positive effect on first-pass success rate and a reduction in chest compression interruptions when using the Glidescope^®^ videolaryngoscope [39]. The positive effect on first-pass success was also seen in manikin studies with a CPR setting, although in lesser amounts. The time needed for intubation during CPR was no longer for the Glidescope^®^ videolaryngoscope in these manikin trials [23,25,27].

Many of the analyzed groups showed significant between-study heterogeneity. This could be a limitation for the interpretation of our results. Differences in the definition of successful and failed intubation existed in the included studies. Failed intubation was defined as intervention by senior staff and/or actual misplacement of the tube. Another explanation could be the difference in initial skill training in the clinicians. Nearly all studies used different approaches to train tracheal intubation clinicians. All manikin studies used training varying from five minutes to one hour, and the clinical studies a training of ten minutes to eight hours. This could also explain differences in success rates between studies and thus significant between-study heterogeneity.

Several limitations of our systematic review should be highlighted. First of all, the number of clinical studies is limited. Furthermore, in our analysis of the manikin studies, subgroups with different scenarios were included. Except for the manikin with a normal airway, all subgroups consisted of no more than three studies. To overcome the small number of studies in each subgroup, we pooled the manikin studies employing difficult airway scenarios as an entire group. Random effects model was employed. As a result, the pooled estimates are more conservative when significant between-study heterogeneity exists [40]. Our systematic review included both clinical and simulation trials with the use of manikins. A manikin can simulate reality only to a limited degree. Despite the resemblances, even the most advanced high-fidelity simulation manikins are unable to fully recreate the feel and finer aspects of human airway anatomy [41]. Especially for clinicians with limited experience, intubation during prehospital resuscitation in an out-of-hospital cardiac arrest airway management can be far more challenging than in manikins. This might explain why clinical trials show a stronger effect in favor of the Glidescope^®^ videolaryngoscopy. More (randomized) clinical studies are needed to confirm the effects, especially in the setting of prehospital CPR. Finally, our approach to focus on one type of videolaryngoscope lead to uncertainty about whether or not the results are generalizable to other types of videolaryngoscopes.

Our findings provide specific insight into tracheal intubation with the Glidescope^®^ videolaryngoscopy by clinicians with limited experience. Previous reviews often include a mix of experience levels and multiple types of videolaryngoscopes [42,43]. Griesdale et al. [43] published a systematic review comparing direct laryngoscopy with Glidescope^®^ videolaryngoscopy. However, the authors included all studies, regardless of the level of experience of the clinicians. The increased success rate with the Glidescope^®^ found in our review is not seen among experienced intubators in the systematic review by Griesdale et al. [43]. In their systematic review, two studies focused on inexperienced personnel; one of those is also included in our systematic review [38], and the other employed nasotracheal intubation which was one of the exclusion criteria in our study [44]. Videolaryngoscopy was also shown to improve the first-pass success rate in emergency intubations in less experienced clinicians in a recent systematic review by Arulkumaran et al. [42]. However, various types of videolaryngoscopes and different operator experience levels for tracheal intubation were included in this review.

The guidelines by the International Liaison Committee on Resuscitation and European Resuscitation Council recommend that tracheal intubation should only be performed by rescuers with a high intubation success rate [45,46]. However, clinicians with limited intubation experience can still be confronted with patients in whom bag-valve-mask ventilation and supraglottic airway device placement are not successful. Especially in the prehospital, remote, or military setting, experienced airway clinicians may take an unacceptably long time to get to the patient. Tracheal intubation is a complex and high-risk procedure, especially when performed by clinicians with limited experience. Large studies on airway management during out-of-hospital cardiac arrest (OHCA) show first-pass success rates of 60–70% [47,48]. Multiple intubation attempts can distract EMS clinicians from ensuring high-quality chest compressions and treating the cause of the arrest. This might be the explanation for a recent study showing that multiple intubation attempts are associated with a decrease in survival [49]. It, therefore, seems important that efforts should be made to improve the tracheal intubation first-pass success rate. When clinicians with limited intubation experience are confronted with a patient requiring tracheal intubation, the use of the Glidescope helps to improve the first-pass success rate, time to intubation, and CPR quality. Videolaryngoscopy is also the intubation technique to be considered in (suspected) COVID-19 patients requiring tracheal intubation [1]. The current European Resuscitation Council Guidelines state that the rescuers' choice of the use of videolaryngoscopy during CPR should be guided by local protocols and rescuer experience [46]. With our review, we hope to provide the evidence needed to consider the use of the Glidescope^®^ videolaryngoscope, particularly during CPR.

## 5. Conclusions

Tracheal intubation performed by clinicians with limited intubation experience (<10 intubations per year) using the Glidescope^®^ videolaryngoscope has a higher first-pass success rate and shorter time to intubation when compared to direct laryngoscopy. Furthermore, intubation using the Glidescope^®^ videolaryngoscope helps to minimize chest compression interruption during CPR. Although the number of clinical studies is limited, the use of the Glidescope^®^ videolaryngoscope by clinicians with limited experience in tracheal intubation has important advantages when other initial airway techniques have failed. In particular, in the setting of prehospital advanced life support, further clinical studies are needed to confirm these findings and determine the effects on outcomes in out-of-hospital cardiac arrest.

## Figures and Tables

**Table 1:** Study characteristics of clinical and manikin trials. (*) The number of intubations in this table includes only the first scenario with normal airway. (ASA = American Society of Anesthesiologists, CL = Cormack–Lehane, CPR = cardiopulmonary resuscitation, DL = direct laryngoscopy, GS = Glidescope^®^ videolaryngoscopy, USA = United States of America).

**Clinical studies**						
**First author, year**	**Country of origin**	**Number of patients**	**Randomized per group**	**Operators**	**Training**	**Patients**
Nouruzi, 2009 [38]	Essen, Germany	200	DL: 100 GS: 100	Students (paramedic, nurse & medical)	Manikin training	ASA 1–2 predicted non difficult airway
Ayoub, 2010 [36]	Beirut, Lebanon	42	DL: 21 GS: 21	Medical students	Manikin training	ASA 1–2 predicted non difficult airway
Hirabayashi, 2010 [37]	Shimotsuke, Japan	200	DL: 100 GS: 100	Non-anaesthesia residents	Short demonstration and 5–6 practices on a manikin	ASA 1–2 predicted non difficult airway
Park, 2015 [39]	Seoul, Republic of Korea	83	DL: 34 GS: 49	Inexperienced emergency physicians	Airway session of 8 h	CPR patients
4		525	DL: 255 GS: 270			
**Manikin studies**						
**First author, year**	**Country of origin**	**Number of intubations**	**Randomized per group**	**Operators**	**Training**	**Scenario**
Lim, 2004 [16]	Singapore	80	DL: 20 GS: 20	Medical students	Instructions and 3 min practice	Normal airway CL grade 3
You, 2009 [20]	Ulsan, Korea	41	DL: 20 GS: 21	Medical students	Lecture of 30 min	Normal airway
Lin, 2009 [17]	Hualien, Taiwan	164	DL: 41 GS: 41	Medical students	Instructions and 5 attempts with both devices	Normal airway CL grade 3
Malik, 2009 [18]	Galway, Ireland	318	DL: 53 * GS: 53 *	Medical students	Instruction of 5 min and 5 attempts	Normal airway Cervical immobilisation Pharyngeal obstruction
Powell, 2009 [19]	Sheffield, UK	42	DL: 21 GS: 21	Non-anaesthesists	Individual standard demonstration	Normal airway
Cinar, 2011 [21]	Ankara, Turkey	242	DL: 121 GS: 121	Paramedic students	Lecture and demonstration	Normal airway
Kaki, 2011 [22]	Jeddah, Saudi Arabia	100	DL: 50 GS: 50	Non-anaesthesists	Standardized instruction and practice	Normal airway
Kim, 2011 [23]	Seoul, Republic of Korea	80	DL: 20 GS: 20	Paramedic students	Training of one hour	Normal airway CPR on the floor
Shin, 2011 [25]	Seoul, Republic of Korea	128	DL: 32 GS: 32	Interns with <10 tracheal intubations	Instruction of 20 min and 3 intubations on a manikin	Normal airway Chest compressions
Stroumpoulis, 2011 [26]	Athens, Greece	88	DL: 44 GS: 44	ACLS providers	Brief presentation and 5 min of practice	Normal airway
Wass, 2011 [24]	Rochester, USA	100	DL: 25 GS: 25	Medical students	Tutorial of 5–10 min	Normal airway Pharyngeal obstruction
Xanthos, 2011 [27]	Athens, Greece	180	DL: 45 GS: 45	Doctors inexperienced in airway management	Instruction of 20 min and practice on manikin	Normal airway Chest compressions
Xanthos, 2012 [28]	Athens, Greece	192	DL: 96 GS: 96	Medical and nursing graduates	Instruction of 20 min and practice on manikin	Normal airway
Biermann, 2013 [29]	Amsterdam, the Netherlands	78	DL: 39 GS: 39	Unexperienced registrars in internal medicine	Explanation and demonstration	Normal airway
Tung, 2013 [30]	Vancouver, Canada	68	DL: 34 GS: 34	Medical students	Standardized video instruction and 10 min of practice	Normal airway
Wang, 2013 [31]	Hualien, Taiwan	120	DL: 20 GS: 20	Medical students	Demonstration of 3–5 min and practice 1–3 times on bodies	Normal airway On the floor Cervical immobilisation Cervical immobilisation on the floor
Ambrosio, 2014 [32]	San Diego, USA	40	DL: 19 GS: 21	First-year non anaesthesia residents	Manikin training, ended upon 1 successfull intubation with both DL and Glidescope®	CL grade 3 + cervical immobilisation
Kim, 2014 [33]	Seoul, Republic of Korea	156	DL: 39 GS: 39	Medical students	5 intubations	Normal airway Cervical immobilisation
Bahhatiq, 2016 [34]	Makkah Mukarramah, Saudi Arabia	200	DL: 50 GS: 50	Paramedic students	Lecture of one hour, demonstration of 10 min and practice one time	Normal airway Cervical immobilisation
Pieters, 2016 [35]	Nijmegen, the Netherlands	130	DL: 65 GS: 65	Medical students	Demonstration of 5 min, no practice	Normal airway
20		2547	DL: 1273 GS: 1274			

**Table 2:** Outcomes of clinical and manikin trials comparing Glidescope^®^ videolaryngoscope to direct laryngoscopy. We created separate rows in the table for several studies as they used different scenarios in the same study. (*) The data in this row include only the first scenario with normal airways in this study. (CL = Cormack–Lehane, sec. = seconds, SD = standard deviation).

	Successful First Intubation Attempt			Time to Intubation (sec.) +/- SD		
First Author, Year	Direct Laryngoscopy	Glidescope^®^	P	Direct Laryngoscopy	Glidescope^®^	P
**Clinical studies**						
Nouruzi, 2009 [38]	51/100 (51.0%)	93/100 (93.0%)	*p* < 0.001	89.0 +/- 35.0	63.0 +/- 30.0	*p* < 0.01
Ayoub, 2010 [36]	3/21 (14.3%)	10/21 (47.6%)	*p* = 0.04	70.7 +/- 7.50	59.3 +/- 4.4	*p* = 0.006
Hirabayashi, 2010 [37]	77/100 (77.0%)	94/100 (94.0%)	*p* = 0.03	72.0 +/- 47.0	64.0 +/- 33.0	*p* = 0.13
Park, 2015 [39]	19/34 (55.9%)	45/49 (91.8%)	*p* < 0.001	62.0 +/- 40.0	37.0 +/- 19.3	*p* < 0.001
**Manikin with normal airway**						
Lim, 2004 [16]	19/20 (95.0%)	20/20 (100%)	*p* = 1	14.5 +/- 5.20	24.5 +/- 18.1	*p* = 0.02
You, 2009 [20]	11/21 (55.0%)	12/20 (57.0%)	*p* > 0.05	30.5 +/- 18.5	26.6 +/- 14.3	*p* = 0.35
Lin, 2009 [17]	36/41 (87.8%)	35/41 (85.3%)	*p* = 1	40.6 +/- 5.30	61.4 +/- 4.80	*p* < 0.001
Malik, 2009 * [18]	49/53 (92.4%)	51/53 (96.2%)	*p* = 0.68	30.7 +/- 16.5	25.1 +/- 15.7	*p* = 0.08
Powell, 2009 [19]	18/20 (90.0%)	20/20 (100%)	*p* = 0.49	20.8 +/- 6.40	26.2 +/- 11.1	*p* = 0.07
Cinar, 2011 [21]	95/121 (78.5%)	111/121 (91.7%)	*p* = 0.006	25.1 +/- 14.0	22.6 +/- 10.0	*p* = 0.11
Kaki, 2011 [22]	37/50 (74.0%)	50/50 (100%)	*p* < 0.001	60.3 +/- 8.40	18.7 +/- 0.40	*p* < 0.001
Kim, 2011 [23]	18/20 (90.0%)	16/20 (80.0%)	*p* = 0.15	18.3 +/- 5.50	21.4 +/- 5.60	*p* = 0.24
Shin, 2011 [25]	31/32 (96.9%)	32/32 (100%)	*p* = 0.36	16.5 +/- 6.70	14.3 +/- 3.90	*p* = 0.03
Stroumpoulis, 2011 [26]	12/44 (27.3%)	29/44 (65.9%)	*p* < 0.001	17.0 +/- 4.00	16.0 +/- 5.00	*p* > 0.05
Wass, 2011 [24]	20/25 (80.0%)	25/25 (100%)	*p* = 0.05	46.0 +/- 43.0	50.0 +/- 33.1	*p* = 0.71
Xanthos, 2011 [27]	33/45 (73.3%)	45/45 (100%)	*p* = 0.001	12.0 +/- 10.4	13.0 +/- 9.60	*p* = 0.64
Xanthos, 2012 [28]	26/96 (27.1%)	28/96 (29.1%)	*p* = 0.87	21.5 +/- 4.80	21.7 +/- 5.50	*p* = 0.79
Biermann, 2013 [29]	27/39 (69.2%)	36/39 (92.3%)	*p* = 0.02	39.0 +/- 12.0	75.0 +/- 40.0	*p* < 0.001
Tung, 2013 [30]	30/34 (88.2%)	34/34 (100%)	*p* = 0.11	17.4 +/- 6.60	17.7 +/- 4.50	*p* = 0.45
Wang, 2013 [31]	20/20 (100%)	19/20 (95.0%)	*p* = 1	20.5 +/- 8.50	21.0 +/- 9.60	*p* = 0.86
Kim, 2014 [33]	34/39 (87.1%)	23/29 (58.9%)	*p* = 0.008	33.2 +/-18.0	26.6 +/- 9.50	*p* = 0.18
Bahhatiq, 2016 [34]	68/100 (68.0%)	91/100 (91.0%)	*p* < 0.001	31.5 +/- 26.0	22.0 +/- 17.8	*p* < 0.001
Pieters, 2016 [35]	51/65 (78.4%)	58/65 (89.2%)	*p* = 0.1	29.3 +/- 12.5	26.0 +/- 25.2	*p* = 0.35
**Manikin with chest compressions**						
Shin, 2011 [25]	24/32 (75.0%)	31/32 (96.9%)	*p* = 0.01	30.1 +/- 26.3	19.2 +/- 11.8	*p* = 0.006
Xanthos, 2011 [27]	20/45 (44.4%)	43/45 (95.6%)	*p* < 0.001	15.0 +/- 13.3	13.0 +/- 9.60	*p* = 0.42
**Manikin with chest compressions on the floor**						
Kim, 2011 [23]	15/20 (75.0%)	17/20 (85.0%)	*p* = 0.69	24.1 +/- 10.4	24.1 +/- 8.90	*p* = 0.99
**Manikin with CL grade 3**						
Lim, 2004 [16]	14/20 (70.0%)	19/20 (95.0%)	*p* = 0.09	156 +/- 241	30.5 +/- 52.8	*p* < 0.001
Lin, 2009 [17]	18/41 (43.9%)	36/41 (87.8%)	*p* < 0.001	98.7 +/- 10.2	64.3 +/- 6.50	*p* < 0.001
**Manikin with cervical immobilisation**						
Malik, 2009 [18]	47/53 (88.7%)	49/53 (92.5%)	*p* = 0.74	38.0 +/- 14.8	23.0 +/- 14.8	*p* < 0.001
Wang, 2013 [31]	20/20 (100%)	17/20 (85.0%)	*p* = 0.23	26.5 +/- 5.90	31.5 +/- 12.6	*p* = 0.12
Kim, 2014 [33]	34/39 (87.1%)	35/39 (89.7%)	*p* = 0.72	26.1 +/- 9.60	23.8 +/- 9.60	*p* = 0.49
Bahhatiq, 2016 [34]	60/100 (60.0%)	88/100 (88.0%)	*p* < 0.001	40.0 +/- 19.3	21.0 +/- 14.8	*p* < 0.001
**Manikin with cervical immobilisation on the floor**						
Wang, 2013 [31]	19/20 (95.0%)	18/20 (90.0%)	*p* = 1	26.0 +/- 7.80	36.0 +/- 8.50	*p* < 0.001
**Manikin with cervical immobilisation and CL grade 3**						
Ambrosio, 2014 [32]	9/19 (47.7%)	21/21 (100%)	*p* < 0.001	69.0 +/- 36.1	23.1 +/- 11.0	*p* < 0.001
**Manikin with pharyngeal obstruction**						
Malik, 2009 [18]	33/53 (62.3%)	48/53 (90.6%)	*p* = 0.001	38.0 +/- 16.3	21.0 +/- 11.1	*p* < 0.001
Wass, 2011 [24]	23/25 (92.0%)	25/25 (100%)	*p* = 0.49	34.0 +/- 23.0	38.0 +/- 30.4	*p* = 0.60
**Manikin on the floor**						
Wang, 2013 [31]	20/20 (100%)	20/20 (100%)	*p* = 1	24.0 +/- 6.70	29.0 +/- 6.30	*p* = 0.05

## Data Availability

Data can be requested via the corresponding author.

## Supplementary Materials

The following supporting information can be downloaded at: https://www.mdpi.com/article/10.3390/jcm11216291/s1, Supplementary Materials 1: PROSPERO Protocol, Supplementary Materials 2: PRISMA Checklist, Supplementary Materials 3: References of Excluded Articles, Supplementary Materials 4: GRADE Table.
