# Supplementary material for: Tracheal Intubation during Advanced Life Support Using Direct Laryngoscopy versus Glidescope® Videolaryngoscopy by Clinicians with Limited Intubation Experience: A Systematic Review and Meta-Analysis
Source: J Clin Med. 2022 Oct 26;11(21):6291. doi: 10.3390/jcm11216291 (PMC9655434; doi:10.3390/jcm11216291)
Supplement: Supplementary file 1 [file jcm-11-06291-s001.zip › Supplementary Materials 1. Supp1 Prospero.pdf]

Citation

Kamil Wojciechowicz, Hans van Schuppen. Direct laryngoscopy vs glidescope video-laryngoscopy by the inexperienced. PROSPERO 2018 CRD42018096251 Available from:
https://www.crd.york.ac.uk/prospero/display_record.php?ID=CRD42018096251

Review question

Succesful intubation by inexperienced intubators using the glidescope videolaryngoscope compared to direct laryngoscopy in the prehospital setting

Searches

MEDLINE/PubMed and Embase was systematically searched (1966 to February 13th, 2018)

Types of study to be included

Randomized controlled trials performed both in humans as well as manikins

Condition or domain being studied

Prehospital patients in need to be intubated

Participants/population

Inexperienced intubators = less than 10 intubations in their career

Intervention(s), exposure(s)

Glidescope videolaryngoscope

Comparator(s)/control

Direct laryngoscopy

Context

comparison of direct laryngoscopy to Glidescope video-laryngoscopy

comparison of direct laryngoscopy to Glidescope Ranger video-laryngoscopy

made reference to either using the Glidescope video-laryngoscope or the Glidescope Ranger video-laryngoscope by inexperienced personnel

Main outcome(s)

successful first-attempt intubation

Additional outcome(s)

time to intubation

Data extraction (selection and coding)

An article was included if they 1) were randomized or quasi-randomized controlled trials, 2) compared direct laryngoscopy to Glidescope video-laryngoscopy, 3) addressed adult patients, 4) contained any outcome of interest (successful first-attempt intubation and/or time to intubation)

The article was excluded if 1) the operator was experienced in intubation, 2) nasotracheal intubation was performed. Disagreement was resolved by discussion and arbitrated if necessary by a third independent researcher.

Risk of bias (quality) assessment

Risk of bias will be assessed using the cochrane revmanager risk of bias tool in which all included articles will be graded between either "low" or "high" and in the case of insufficient information "unclear"

Strategy for data synthesis

a quantitative synthesis will be used with relative risk (RR) as the summary measure for the successful first intubation outcome and the mean difference (MD), in seconds, as the summary measure for time to intubate. The random effects method of Mantel-Haenszel was used to generate a pooled RR or WMD across studies. We assessed statistical heterogeneity using Cochran’s Q statistic (with P<0,05 considered significant) and expressed the quantity using the I^2 statistic and 95% confidence interval (CI). We followed the cochrane handbook classification for I^2 where 0-40% represents “might not be important heterogeneity,” 30-60% “may represent moderate heterogeneity,” 50-90% “may represent substantial heterogeneity” and 75-100%

Analysis of subgroups or subsets
The data for intubations on patients will be analyzed separate from the data extracted from studies performed on manikins. There will also be subgroups for "difficult" intubations e.g. cormack-lehane 3, oropharyngeal swelling and or neck stabilization during intubation

Contact details for further information
Kamil Wojciechowicz
k.wojciechowicz@amc.nl

Organisational affiliation of the review
Academisch Medisch Centrum, Amsterdam
www.amc.nl

Review team members and their organisational affiliations
Mr Kamil Wojciechowicz. AMC
Mr Hans van Schuppen. AMC

Type and method of review
Intervention

Anticipated or actual start date
18 August 2017

Anticipated completion date
18 August 2018

Funding sources/sponsors
none

Conflicts of interest

Language
English

Country
Netherlands

Stage of review
Review Ongoing

Subject index terms status
Subject indexing assigned by CRD

Subject index terms
Humans; Intubation, Intratracheal; Laryngoscopes; Laryngoscopy

Date of registration in PROSPERO
23 July 2018

Date of first submission
10 May 2018

Details of any existing review of the same topic by the same authors
non

Stage of review at time of this submission

Stage	Started	Completed
Preliminary searches	Yes	No
Piloting of the study selection process	Yes	No
Formal screening of search results against eligibility criteria	Yes	No
Data extraction	Yes	No
Risk of bias (quality) assessment	Yes	No
Data analysis	Yes	No

The record owner confirms that the information they have supplied for this submission is accurate and complete and they understand that deliberate provision of inaccurate information or omission of data may be construed as scientific misconduct.
The record owner confirms that they will update the status of the review when it is completed and will add publication details in due course.

Versions
23 July 2018
