# Supplementary material for: Tracheal Intubation during Advanced Life Support Using Direct Laryngoscopy versus Glidescope® Videolaryngoscopy by Clinicians with Limited Intubation Experience: A Systematic Review and Meta-Analysis
Source: J Clin Med. 2022 Oct 26;11(21):6291. doi: 10.3390/jcm11216291 (PMC9655434; doi:10.3390/jcm11216291)
Supplement: Supplementary file 1 [file jcm-11-06291-s001.zip › Supplementary Materials 3. Excluded articles.pdf]

Supplemental material 3 – References of excluded articles [1-55]

[1] Hypes CD, Stolz U, Sakles JC, Joshi RR, Natt B, Malo J, et al. Video Laryngoscopy Improves Odds of First-Attempt Success at Intubation in the Intensive Care Unit. A Propensity-matched Analysis. Annals of the American Thoracic Society. 2016;13:382-90.
[2] Janz DR, Semler MW, Lentz RJ, Matthews DT, Assad TR, Norman BC, et al. Randomized Trial of Video Laryngoscopy for Endotracheal Intubation of Critically Ill Adults. Critical care medicine. 2016;44:1980-7.
[3] Joshi R, Hypes CD, Greenberg J, Snyder L, Malo J, Bloom JW, et al. Difficult Airway Characteristics Associated with First-Attempt Failure at Intubation Using Video Laryngoscopy in the Intensive Care Unit. 2017;14:368-75.
[4] Kamal S, Ali QE, Amir SH, Ahmed S, Pal K. King Vision video laryngoscope versus Lightwand as an intubating device in adult patients with Mallampatti grade III and IV patients. Journal of clinical anesthesia. 2016;34:483-9.
[5] Kim JW, Lee KR, Hong DY, Baek KJ, Lee YH, Park SO. Efficacy of various types of laryngoscope (direct, Pentax Airway Scope and GlideScope) for endotracheal intubation in various cervical immobilisation scenarios: a randomised cross-over simulation study. BMJ open. 2016;6:e011089.
[6] Kim JW, Park SO, Lee KR, Hong DY, Baek KJ, Lee YH, et al. Video laryngoscopy vs. direct laryngoscopy: Which should be chosen for endotracheal intubation during cardiopulmonary resuscitation? A prospective randomized controlled study of experienced intubators. Resuscitation. 2016;105:196-202.
[7] Maldini B, Hodzovic I, Goranovic T, Mesaric J. CHALLENGES IN THE USE OF VIDEO LARYNGOSCOPES. Acta clinica Croatica. 2016;55 Suppl 1:41-50.
[8] Misirlioglu G, Sen O. Comparison of GlideScope video laryngoscopy and Macintosh laryngoscope in ear-nose and throat surgery. Irish journal of medical science. 2016;185:729-33.
[9] Naito H, Guyette FX, Martin-Gill C, Callaway CW. Video Laryngoscopic Techniques Associated with Intubation Success in a Helicopter Emergency Medical Service System. Prehospital emergency care : official journal of the National Association of EMS Physicians and the National Association of State EMS Directors. 2016;20:333-42.
[10] Parasa M, Yallapragada SV, Vemuri NN, Shaik MS. Comparison of GlideScope video laryngoscope with Macintosh laryngoscope in adult patients undergoing elective surgical procedures. Anesthesia, essays and researches. 2016;10:245-9.
[11] Sakles JC, Corn GJ, Hollinger P, Arcaris B, Patanwala AE, Mosier JM. The Impact of a Soiled Airway on Intubation Success in the Emergency Department When Using the GlideScope or the Direct Laryngoscope. Academic emergency medicine : official journal of the Society for Academic Emergency Medicine. 2017;24:628-36.
[12] Sakles JC, Douglas MJK, Hypes CD, Patanwala AE, Mosier JM. Management of Patients with Predicted Difficult Airways in an Academic Emergency Department. The Journal of emergency medicine. 2017.
[13] Schroder H, Zoremba N, Rossaint R, Deusser K, Stoppe C, Coburn M, et al. Intubation performance using different laryngoscopes while wearing chemical protective equipment: a manikin study. BMJ open. 2016;6:e010250.
[14] Trimmel H, Kreutziger J, Fitzka R, Szuts S, Derdak C, Koch E, et al. Use of the GlideScope Ranger Video Laryngoscope for Emergency Intubation in the Prehospital Setting: A Randomized Control Trial. Critical care medicine. 2016;44:e470-6.

[15] Vargas M, Pastore A, Aloj F, Laffey JG, Servillo G. A comparison of videolaryngoscopes for tracheal intubation in predicted difficult airway: a feasibility study. BMC anesthesiology. 2017;17:25.
[16] Yousif S, Machan JT, Alaska Y, Suner S. Airway Management in Disaster Response: A Manikin Study Comparing Direct and Video Laryngoscopy for Endotracheal Intubation by Prehospital Providers in Level C Personal Protective Equipment. Prehospital and disaster medicine. 2017:1-5.
[17] Yumul R, Elvir-Lazo OL, White PF, Sloninsky A, Kaplan M, Kariger R, et al. Comparison of three video laryngoscopy devices to direct laryngoscopy for intubating obese patients: a randomized controlled trial. Journal of clinical anesthesia. 2016;31:71-7.
[18] Huang HB, Peng JM, Xu B, Liu GY, Du B. Video Laryngoscopy for Endotracheal Intubation of Critically Ill Adults: A Systemic Review and Meta-analysis. Chest. 2017.
[19] Lee JK, Kang H, Choi HJ. Changes in the first-pass success rate with the GlideScope video laryngoscope and direct laryngoscope: a ten-year observational study in two academic emergency departments. Clinical and experimental emergency medicine. 2016;3:213-8.
[20] Aleksandrowicz S, Szarpak L. A comparison of GlideScope and Macintosh laryngoscopes for endotracheal intubation performed by nurses. The American journal of emergency medicine. 2016;34:2041.
[21] Schulte TE, Ringenberg KJ, Lisco SJ, Sayles H, Shillcutt SK. Trainee experience and success of urgent airway management. Journal of clinical anesthesia. 2016;35:536-42.
[22] Jarman AF, Hopkins CL, Hansen JN, Brown JR, Burk C, Youngquist ST. Advanced Airway Type and Its Association with Chest Compression Interruptions During Out-of-Hospital Cardiac Arrest Resuscitation Attempts. Prehospital emergency care : official journal of the National Association of EMS Physicians and the National Association of State EMS Directors. 2017:1-8.
[23] Lascarrou JB, Boisrame-Helms J, Bailly A, Le Thuaut A, Kamel T, Mercier E, et al. Video Laryngoscopy vs Direct Laryngoscopy on Successful First-Pass Orotracheal Intubation Among ICU Patients: A Randomized Clinical Trial. Jama. 2017;317:483-93.
[24] Parotto M, Niazi A, Wong DT, Cooper RM, Yang GZ, Xue FS, et al. Comparing video and direct laryngoscope for endotracheal intubation during CPR. Canadian journal of anaesthesia = Journal canadien d'anesthesie. 2017;35:602-3.
[25] Wesley K, Wesley K. QUALITY OF VIEW. Video laryngoscopy improves intubation success rate. JEMS : a journal of emergency medical services. 2016;41:22.
[26] Wallace MC, Britton ST, Meek R, Walsh-Hart S, Carter CTE, Lisco SJ. Comparison of five video-assisted intubation devices by novice and expert laryngoscopists for use in the aeromedical evacuation environment. Military Medical Research. 2017;4:20.
[27] Abola RE, Bennett-Guerrero E. Video Laryngoscopy Versus Direct Laryngoscopy in the ICU: Don't Throw Away That MAC Blade Just Yet. Critical care medicine. 2016;44:2106-7.
[28] Drozd A, Madziala M. Comparison of video laryngoscopy and Macintosh direct laryngoscopy for airway management. The American journal of emergency medicine. 2016;34:1886.
[29] Loh NW, Tan J. Intubation With Video Laryngoscopy vs Direct Laryngoscopy. Jama. 2017;317:2129-30.
[30] O'Gara B, Brown S, Talmor D. Video Laryngoscopy in the Intensive Care Unit: Seeing Is Believing, But That Does Not Mean It's True. Jama. 2017;317:479-80.
[31] Kuwahara S, Goto T, Chhavi S, Abhyuday K, Parin L. Video laryngoscope as the new standard of care in trauma ED. PloS one. 2016;34:1313-4.
[32] MacDonald RD. Articles That May Change Your Practice: Video Laryngoscopy. Air medical journal. 2016;35:114-6.

[33] Mendelson JS, Felner KJ, Kaufman BS. Randomized Trial of Video Laryngoscopy for Endotracheal Intubation of Critically Ill Adults: More Data, More Questions. Critical care medicine. 2017;45:e460-e1.
[34] Saddawi-Konefka D, Baker KH, Wiener-Kronish JP. Intubation With Video Laryngoscopy vs Direct Laryngoscopy. Jama. 2017;317:2130-1.
[35] Zhao BC, Huang TY, Liu KX. Video laryngoscopy for ICU intubation: a meta-analysis of randomised trials. Intensive care medicine. 2017;43:947-8.
[36] Xue FS, Liu GP, Sun C, Li RP. Comparing Emergency Intubation with Direct and Video Laryngoscopy. Academic emergency medicine : official journal of the Society for Academic Emergency Medicine. 2016;23:747-8.
[37] Xue FS, Liu YY, Li HX. Intubation With Video Laryngoscopy vs Direct Laryngoscopy. Jama. 2017;317:2130.
[38] Xue FS, Liu YY, Li HX, Yang GZ. Is video laryngoscopy really superior to direct laryngoscopy for emergency intubation in prehospital trauma patients? Internal and emergency medicine. 2017;12:139-40.
[39] Xue FS, Yang GZ, Sun C. Performance of GlideScope Ranger Video Laryngoscope for Prehospital Emergency Intubation. Critical care medicine. 2016;44:e1141.
[40] Heuer JF, Heitmann S, Crozier TA, Bleckmann A, Quintel M, Russo SG. A comparison between the GlideScope(R) classic and GlideScope(R) direct video laryngoscopes and direct laryngoscopy for nasotracheal intubation. Journal of clinical anesthesia. 2016;33:330-6.
[41] Jin HY, Xue FS, Yang GZ. Controlling tip of nasotracheal tube under video laryngoscopy. Journal of anesthesia. 2016;30:917.
[42] Mahran EA, Hassan ME. Comparative randomised study of GlideScope(R) video laryngoscope versus flexible fibre-optic bronchoscope for awake nasal intubation of oropharyngeal cancer patients with anticipated difficult intubation. Indian journal of anaesthesia. 2016;60:936-8.
[43] Arslan ZI, Turna C, Gumus NE, Toker K, Solak M. Intubation of a Paediatric Manikin in Tongue Oedema and Face-to-Face Simulations by Novice Personnel: a Comparison of Glidescope, Airtraq and Direct Laryngoscopy. Turkish journal of anaesthesiology and reanimation. 2016;44:71-5.
[44] Balaban O, Hakim M, Walia H, Tumin D, Lind M, Tobias JD. A Comparison of Direct Laryngoscopy and Videolaryngoscopy for Endotracheal Intubation by Inexperienced Users: A Pediatric Manikin Study. Pediatric emergency care. 2017.
[45] Eisenberg MA, Green-Hopkins I, Werner H, Nagler J. Comparison Between Direct and Video-assisted Laryngoscopy for Intubations in a Pediatric Emergency Department. Academic emergency medicine : official journal of the Society for Academic Emergency Medicine. 2016;23:870-7.
[46] Grunwell JR, Kamat PP, Miksa M, Krishna A, Walson K, Simon D, et al. Trend and Outcomes of Video Laryngoscope Use Across PICUs. Pediatric critical care medicine : a journal of the Society of Critical Care Medicine and the World Federation of Pediatric Intensive and Critical Care Societies. 2017.
[47] Hippard HK, Kalyani G, Olutoye OA, Mann DG, Watcha MF. A comparison of the Truview PCD and the GlideScope Cobalt AVL video-laryngoscopes to the Miller blade for successfully intubating manikins simulating normal and difficult pediatric airways. Paediatric anaesthesia. 2016;26:613-20.
[48] Karisik M, Janjevic D, Sorbello M. FIBEROPTIC BRONCHOSCOPY VERSUS VIDEO LARYNGOSCOPY IN PEDIATRIC AIRWAY MANAGEMENT. Acta clinica Croatica. 2016;55 Suppl 1:51-4.

[49] Morimoto Y, Ohyamaguchi A, Inoue M, Yokoe C, Hanamoto H, Imaizumi U, et al. Airway management for glossopexy in infants with micrognathia and obstructive breathing. Journal of clinical anesthesia. 2017;36:127-32.
[50] Nair S, Thomas EJ, Katakam L. Video Laryngoscopy vs. Direct Laryngoscopy in Teaching Neonatal Endotracheal Intubation: A Simulation-Based Study. Cureus. 2017;9:e962.
[51] Pallin DJ, Dwyer RC, Walls RM, Brown CA, 3rd. Techniques and Trends, Success Rates, and Adverse Events in Emergency Department Pediatric Intubations: A Report From the National Emergency Airway Registry. Annals of emergency medicine. 2016;67:610-5.e1.
[52] Parker MM, Nuthall G, Brown C, 3rd, Biagas K, Napolitano N, Polikoff LA, et al. Relationship Between Adverse Tracheal Intubation Associated Events and PICU Outcomes. Pediatric critical care medicine : a journal of the Society of Critical Care Medicine and the World Federation of Pediatric Intensive and Critical Care Societies. 2017;18:310-8.
[53] Sinha R, Sharma A, Ray BR, Kumar Pandey R, Darlong V, Punj J, et al. Comparison of the Success of Two Techniques for the Endotracheal Intubation with C-MAC Video Laryngoscope Miller Blade in Children: A Prospective Randomized Study. Anesthesiology research and practice. 2016;2016:4196813.
[54] Sola C, Saour AC, Macq C, Bringuier S, Raux O, Dadure C. Children with challenging airways: What about GlideScope(R) video-laryngoscopy? Anaesthesia, critical care & pain medicine. 2016.
[55] Szarpak L, Truszewski Z, Czyzewski L, Gaszynski T, Rodriguez-Nunez A. A comparison of the McGrath-MAC and Macintosh laryngoscopes for child tracheal intubation during resuscitation by paramedics. A randomized, crossover, manikin study. The American journal of emergency medicine. 2016;34:1338-41.
