# Supplementary material for: Tracheal Intubation during Advanced Life Support Using Direct Laryngoscopy versus Glidescope® Videolaryngoscopy by Clinicians with Limited Intubation Experience: A Systematic Review and Meta-Analysis
Source: J Clin Med. 2022 Oct 26;11(21):6291. doi: 10.3390/jcm11216291 (PMC9655434; doi:10.3390/jcm11216291)
Supplement: Supplementary file 1 [file jcm-11-06291-s001.zip › Supplementary Materials 4. GRADE table.pdf]

Author(s): Hans van Schuppen, Kamil Wojciechowicz, Markus Hollmann, Benedikt Preckel
Question: Glidescope® videolaryngoscopy compared to direct laryngoscopy for endotracheal intubation by providers with limited intubation experience during advanced life support
Setting: cardiopulmonary resuscitation
Bibliography:

Certainty assessment							№ of patients		Effect		Certainty	Importance
№ of studies	Study design	Risk of bias	Inconsistency	Indirectness	Imprecision	Other considerations	Glidescope® videolaryngoscopy	direct laryngoscopy	Relative (95% CI)	Absolute (95% CI)		
**First-pass success rate - clinical studies**												
4	randomised trials	not serious[a]	serious[b]	not serious	not serious	none	242/270 (89.6%)	150/255 (58.8%)	**RR 1.61** (1.16 to 2.23)	**359 more per 1.000** (from 94 more to 724 more)	⊕⊕⊕◯ Moderate	CRITICAL
**First-pass success rate - overall results of all manikin studies**												
20	randomised trials	not serious[a]	serious[b]	serious[c]	not serious	none	1202/1383 (86.9%)	992/1393 (71.2%)	**RR 1.17** (1.09 to 1.25)	**121 more per 1.000** (from 64 more to 178 more)	⊕⊕◯◯ Low	CRITICAL
**First-pass success rate - manikin studies with normal airway scenario**												
19	randomised trials	not serious[a]	serious[b]	serious[c]	not serious	none	735/874 (84.1%)	636/885 (71.9%)	**RR 1.13** (1.05 to 1.21)	**93 more per 1.000** (from 36 more to 151 more)	⊕⊕◯◯ Low	CRITICAL
**First-pass success rate - manikin studies with chest compressions**												
3	randomised trials	not serious[a]	serious[b]	serious[c]	not serious	none	91/97 (93.8%)	59/97 (60.8%)	**RR 1.45** (1.00 to 2.10)	**274 more per 1.000** (from 0 fewer to 669 more)	⊕⊕◯◯ Low	CRITICAL
**First-pass success rate - manikin studies with difficult airway scenario**												
8	randomised trials	not serious[a]	serious[b]	serious[c]	not serious	none	376/412 (91.3%)	297/411 (72.3%)	**RR 1.20** (1.03 to 1.40)	**145 more per 1.000** (from 22 more to 289 more)	⊕⊕◯◯ Low	CRITICAL
**Mean intubation times - clinical studies**												
4	randomised trials	not serious[a]	serious[b]	not serious	not serious	none	270	255	-	MD **17.04 lower** (25.57 lower to 8.51 lower)	⊕⊕⊕◯ Moderate	CRITICAL
**Mean intubation times - overall results of all manikin studies**												
20	randomised trials	not serious[a]	very serious[d]	serious[c]	not serious	none	1393	1394	-	MD **4.11 lower** (9.54 lower to 1.32 higher)	⊕◯◯◯ Very low	CRITICAL
**Mean intubation times - manikin studies with normal airway scenario**												
19	randomised trials	not serious[a]	serious[d]	serious[c]	not serious	none	884	885	-	MD **0.11 higher** (6.84 lower to 7.07 higher)	⊕⊕◯◯ Low	CRITICAL
**Mean intubation times - manikin studies with chest compressions**												
3	randomised trials	not serious[a]	not serious	serious[c]	not serious	none	97	97	-	MD **2.92 lower** (7.82 lower to 1.98 higher)	⊕⊕⊕◯ Moderate	CRITICAL
**Mean intubation times - manikin studies with difficult airway scenario**												
8	randomised trials	not serious[a]	very serious[d]	serious[c]	not serious	none	412	412	-	MD **12.51 lower** (23.56 lower to 1.46 lower)	⊕◯◯◯ Very low	CRITICAL

**Chest compression interruption during intubation**												
1	randomised trials	not serious[a]	serious[e]	not serious	not serious	none	49	34	-	MD **7 lower** (0 to 0)	⨁⨁⨁◯ Moderate	CRITICAL

CI: confidence interval; **MD:** mean difference; **RR:** risk ratio

Explanations

a. Blinding of participants and personnel was not possible, so performance bias cannot be excluded. However, we estimate that this has little effect on outcomes, because first-pass success, intubation time and interruptions are objective outcome measures.
b. There is substantial between-study heterogeneity
c. Indirectness is possible due to the difference in context of a manikin study, compared to actual clinical settings with a patient in cardiac arrest
d. There is a large between-study heterogeneity, including results in different directions
e. Only one study
